# A systematic review for the antidepressant effects of sleep deprivation with repetitive transcranial magnetic stimulation

**DOI:** 10.1186/s12888-015-0674-8

**Published:** 2015-11-14

**Authors:** Qing Tang, Guangming Li, Anguo Wang, Tao Liu, Shenggang Feng, Zhiwei Guo, Huaping Chen, Bin He, Morgan A. McClure, Jun Ou, Guoqiang Xing, Qiwen Mu

**Affiliations:** Department of Radiology & Imaging Institute of Rehabilitation and Development of Brain Function, North Sichuan Medical University Nanchong Central Hospital, 97 South Renmin Road, Shunqing District, Nanchong, 637000 Sichuan China; Department of Oncology, North Sichuan Medical University Nanchong Central Hospital, 97 South Renmin Road, Shunqing District, Nanchong, 637000 Sichuan China; Department of Urology Surgery, North Sichuan Medical University Nanchong Central Hospital, 97 South Renmin Road, Shunqing District, Nanchong, 637000 Sichuan China; Department of Cardiology, North Sichuan Medical University Nanchong Central Hospital, 97 South Renmin Road, Shunqing District, Nanchong, 637000 Sichuan China; Department of Nephrology, North Sichuan Medical University Nanchong Central Hospital, 97 South Renmin Road, Shunqing District, Nanchong, 637000 Sichuan China; Lotus Biotech.com LLC., John Hopkins University-MCC, 9601 Medical Center Drive, Rockville, MD 20850 USA; Peking University Third Hospital, 49 Garden North Road, Haidian District, Beijing, 100080 China

**Keywords:** Depression, Sleep deprivation, Repetitive transcranial magnetic stimulation (rTMS), Systematic review

## Abstract

**Background:**

Sleep deprivation (SD) and repetitive transcranial magnetic stimulation (rTMS) have been commonly used to treat depression. Recent studies suggest that co-therapy with rTMS and SD may produce better therapeutic effects than either therapy alone. Therefore, this study was to review the current findings to determine if rTMS can augment the therapeutic effects of SD on depression.

**Methods:**

Embase, JSTOR, Medline, PubMed, ScienceDirect, and the Cochrane Central Register of Controlled Trials were searched for clinical studies published between January 1985 and March 2015 using the search term “rTMS/repetitive transcranial magnetic stimulation AND sleep deprivation AND depress*”. Only randomized and sham-controlled trials (RCTs) involving the combined use of rTMS and SD in depression patients were included in this systematic review. The scores of the Hamilton Rating Scale for Depression were extracted as primary outcome measures.

**Results:**

Three RCTs with 72 patients that met the inclusion criteria were included for the systematic review. One of the trials reported skewed data and was described alone. The other two studies, which involved 30 patients in the experimental group (SD + active rTMS) and 22 patients in the control group (SD + sham rTMS), reported normally distributed data. The primary outcome measures showed different results among the three publications: two of which showed great difference between the experimental and the control subjects, and the other one showed non-significant antidepressant effect of rTMS on SD. In addition, two of the included studies reported secondary outcome measures with Clinical Global Impression Rating Scale and a self-reported well-being scale which presented good improvement for the depressive patients in the experiment group when compared with the control. The follow-up assessments in two studies indicated maintained results with the immediate measurements.

**Conclusions:**

From this study, an overview of the publications concerning the combined use of rTMS and SD is presented, which provides a direction for future research of therapies for depression. More studies are needed to confirm whether there is an augmentative antidepressant effect of rTMS on SD.

**Electronic supplementary material:**

The online version of this article (doi:10.1186/s12888-015-0674-8) contains supplementary material, which is available to authorized users.

## Background

Depression is a major psychiatric disorder that affects people of all age groups. According to the World Health Organization [1], more than 350 million people suffer from depression worldwide. People with depression have a poor quality of life with limited social and occupational functions, which causes enormous social and economic burdens to the patients’ families and to society. In addition, the suicide rate of refractory depression patients is high (approximately 15 %) [2]. The treatment choices for depression include multiple options, such as pharmacotherapy, psychotherapy, electroconvulsive therapy, etc. These therapies can be used alone or in combination. However, not all depressed patients respond equally well to these therapies. For the majority of patients, depression remains intractable. In fact, there is currently neither a complete cure for depression nor sufficient understanding of the complex pathological mechanism underlying depression at present.

Sleep deprivation (SD) has been applied to treat depression since the 1970s [3]. SD is a powerful antidepressant strategy, and it works by depriving the patients of normal sleep, either totally or partially. Research has shown that SD produces a marked effect within hours in 40 to 60 % of depressed patients [4]. Now, SD has become a common protocol for treating depression. In the recent fMRI study reported by Bosch et al. [5], SD reduced functional connectivity between the posterior cingulate cortex and the bilateral anterior cingulate cortex but enhanced connectivity between the dorsal nexus and the distinct areas in right dorsolateral prefrontal cortex (DLPFC). Moreover, other studies showed that SD was associated with altered neurotransmitter receptor sensitivity and neuroendocrine reactivity [6–8].

In spite of its fast therapeutic effect on depression, SD therapy alone has limitations. The duration of SD’s therapeutic effect is often short-lived, and the depressive symptoms may return soon after the SD treatment is stopped. Hemmeter et al. [4] showed that more than 80 % of the patients who responded to SD relapsed into depression after one night of recovery. In light of its transient effect, different strategies have been tested to augment and prolong the antidepressant effects of SD. Benedetti et al. [9] reported that lithium carbonate could prolong the antidepressant effect of SD. Wu et al. [10] reported that the addition of three non-invasive, circadian-related interventions to SD therapy in bipolar patients who had been medicated could accelerate and maintain the antidepressant response which provided a quick, safe, and sustainable treatment strategy.

Transcranial magnetic stimulation (TMS) is a newly developed non-invasive therapy that was first reported by Barker et al. in 1985 [11, 12]. TMS causes depolarization or hyperpolarization of neurons in the brain. Soon after its invention, a more advanced form of TMS, i.e., repetitive TMS (rTMS), was developed as a treatment for various neurological and psychiatric disorders, including depression, Parkinson’s disease, stroke, and tinnitus. Numerous clinical trials of rTMS therapy have been conducted in depressive patients. Certain types of rTMS with specific parameters have been effective for depressive patients [13–22]. In fact, rTMS was approved in 2008 as a therapeutic option for treatment-resistant major depressive disorders by the U.S. Food and Drug Administration [23]. Although the mechanism is still unknown, rTMS is thought to exert its therapeutic effect on depression by influencing subgenual anterior cingulate functional connectivity [24, 25], brain networks [25], neurotransmitters [26], neuroendocrine effects [27], and neuronal plasticity [28].

Researchers have shown that rTMS can augment or sustain the therapeutic effects of SD in patients with depression, although it has not yet been firmly established how rTMS exerts its augmenting effect on SD therapy. Krstić and Ilić [29] reported that a combination of slow-rate rTMS and partial SD (PSD) had strong synergistic effects on depression. Several randomized and sham-controlled trials (RCTs) have investigated the contributions of rTMS to SD treatment in depression with varying outcomes [30–34]. However, single trials alone usually lack persuasive power due to small sample sizes. Because guidance is needed for the clinical application of rTMS-SD co-therapy, a detailed analysis of the current findings is highly desirable. The goal of this study was to systematically review and analyze the published RCTs involving rTMS-SD co-therapy for patients with depression.

## Methods

This systematic review of rTMS and SD co-therapy for depression was conducted in accordance with the Preferred Reporting Items for Systematic Reviews and Meta-Analyses statement (Additional file 1) as well as with the explanation and elaboration document that evaluates health care interventions [35–37].

### Search strategy

Embase, JSTOR, Medline, PubMed, ScienceDirect, and the Cochrane Central Register of Controlled Trials were searched to identify relevant studies. The search term was “rTMS/repetitive transcranial magnetic stimulation AND sleep deprivation AND depress*”. Because rTMS was first proposed in 1985, the searches were limited to human studies published between January 1985 and March 2015. There was no restriction with respect to language in the searches.

### Study selection

The full text of the articles that appeared to be relevant was carefully checked by two reviewers independently. Publications that met the following criteria were included in the systematic review [38]:

1. The study was an RCT, and the number of participants was no less than five in each arm.
2. The subjects were between 18 and 75 years of age and were diagnosed as having major depression, bipolar disorder, or another form of depression according to the Diagnostic and Statistical Manual of Mental Disorders, 4th Edition (DSM-IV) [39] or Mini-International Neuropsychiatric Interview Criteria (MINI) [40], without any pseudo-depression, history of hysteria or mania, alcoholics, adreno-cortico-tropic-hormone abnormality, hormonal impairment, congenital anomalies, or intake of any anti-depressants.
3. The treatment protocol was that the subjects received active or sham rTMS over the left or right DLPFC as an added treatment to SD.
4. The outcome measures were reported (continuous scales) and evaluated with the Hamilton Rating Scale for Depression (HRSD) [41], Clinical Global Impression Rating Scale (CGI) [42], Montgomery-Åsberg Depression Scale (MADRS) [43], or other scales for depression.

Studies were excluded if they had the following characteristics:

1. The study enrolled subjects who also had other severe neurologic or psychiatric diseases, who had a history of seizures or brain surgery, or who were implanted with electronic devices.
2. Same study subjects were enrolled in other reports.
3. The SD and rTMS treatments were started in conjunction with a new antidepressant medication.
4. The response data could not be obtained for the study even after the authors were contacted.

### Assessment of bias in the included studies

To evaluate the quality of the included studies, the risk of bias assessment tool in Review Manager Software version 5.2 (Cochrane Collaboration, Oxford, England) was used to assess the random sequence generation (selection bias), allocation concealment (selection bias), blinding of participants and personnel (performance bias), blinding of outcome assessment (detection bias), incomplete outcome data (attrition bias), selective reporting (reporting bias), and other biases. The assessment of bias was performed in accordance with the relevant descriptions in the *Cochrane Handbook for Systematic Reviews of Interventions* [44]. If there was sufficient detail to support a judgment for each bias, they were assigned as low risk or high risk; otherwise, they were assigned as unclear risk.

### Data extraction

The data were individually extracted by two reviewers: the first author and another co-author. The data were recorded as follows:

1. Participant characteristics: including the number of subjects, mean age, gender, treatment strategy (i.e., SD + rTMS), primary diagnosis, and presence of pharmacotherapy.
2. rTMS parameters: including stimulation frequency, intensity, sessions, total pulses, coil, stimulating position, and the method of sham rTMS.
3. Outcome measures: HRSD scores were extracted as the primary outcome measure. The scores obtained from the other scales were defined as secondary outcome measures. Both the HRSD scores and the other scores were assessed at the endpoint of intervention and in follow-up.
4. Adverse events: the number and type of adverse event, if reported.

### Review of primary outcome measures

The primary outcome measures (HRSD scores) in the included studies were reviewed. The HRSD scores were calculated based on a multiple item questionnaire which was designed for an indication of depression, mainly including mood, feelings of guilt, suicide, insomnia, work and activities, retardation, agitation, anxiety, hypochondriasis, weight loss, somatic symptoms, and insight [45]. Data were described as mean ± standard deviation (s.d.). If graphs were reported instead of the original data in the articles, data were extracted with the assistance of GetData Graph Digitizer 2.25 (http://getdata-graph-digitizer.com/) based on the *Cochrane Handbook for Systematic Reviews of Interventions* [44]. It is important to note that skewed data could not be expressed as mean ± s.d.. They were usually reported with the means, medians, and inter-quartile ranges as well as the maximums and minimums. The authors of the included articles were contacted for more detailed information about their studies.

### Review of secondary outcome measures

Apart from the HRSD score, other scales such as the CGI and a self-reported well-being scale (BfS), were used to measure the degree of depression. CGI is a 7-point scale commonly used in psychiatry, measuring the illness severity, global improvement or change, and the therapeutic response from subjective aspects, as it requires the surveyor to compare the subjects with typical patients in clinics [41]. CGI is usually used to measure depressive symptoms [46, 47]. BfS is a self-rating scale for the measurement of subjective well-being conditions. It is especially suitable to assess the rapid mood changes [48, 49]. The CGI scores and BfS scores were defined as secondary outcome measures and systematically reviewed for a supplementary proof.

## Results

### Selection and inclusion of studies

**Fig. 1:**
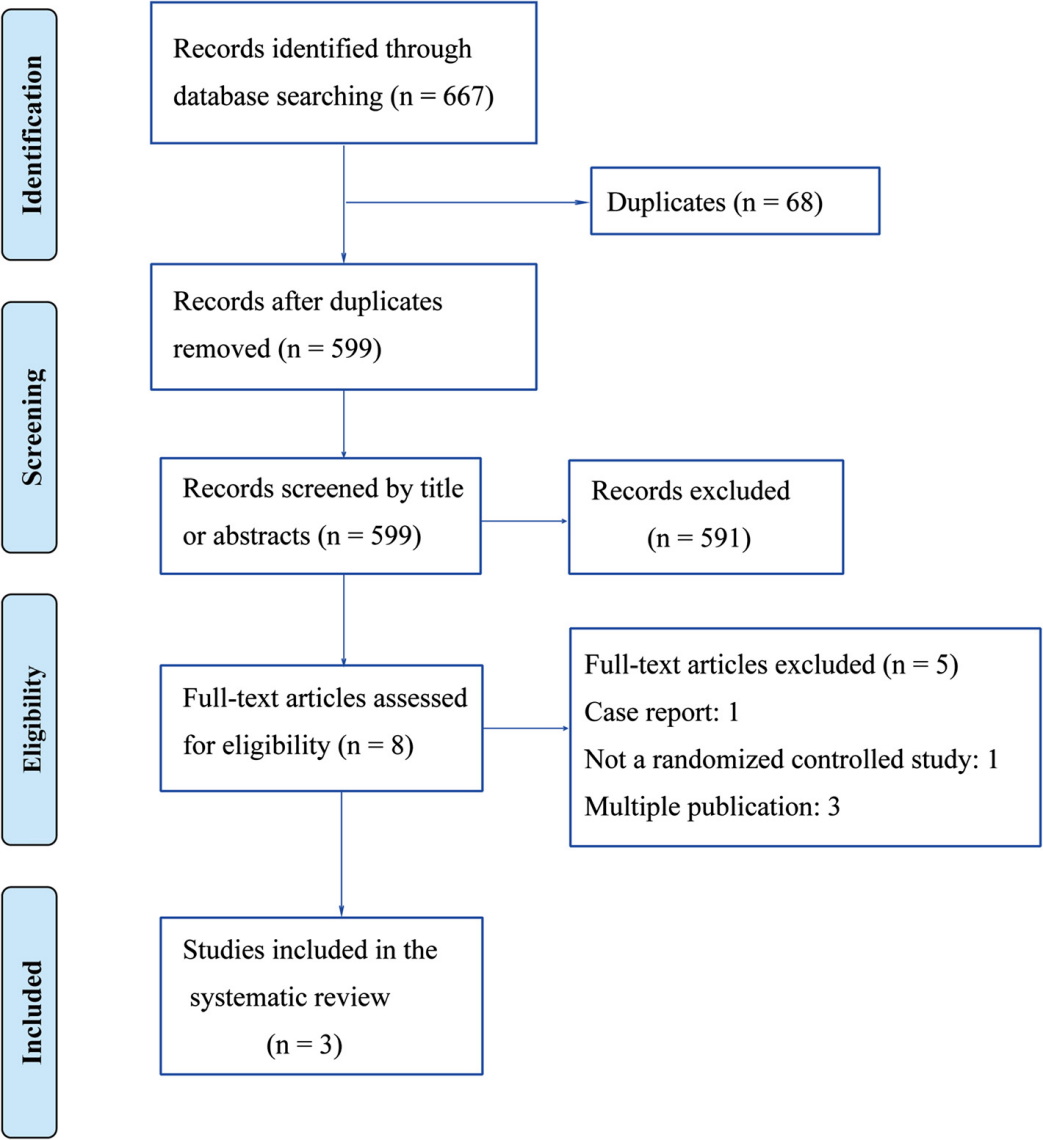
Flow diagram of study selection. Doi: 10.1371/journal.pmed.1000100.g002

The original searches yielded 20 results in Embase, 29 in JSTOR, 15 in Medline, 30 in PubMed, 568 in ScienceDirect, and five in the Cochrane Central Register of Controlled Trials. After exclusion, eight potentially relevant abstracts or articles were obtained based on the title and abstract [29–34, 50]. Of these eight publications, five were excluded for the following reasons: (a) one of them was a case report [29]; (b) one was not an RCT [50]; and (c) three trials contained the same study population as other published studies [31, 34, 51]. The remaining three studies [30, 32, 33] met the inclusion criteria described above and were therefore included in this systematic review. Skewed data were found in one study [30]. The data in the other two studies were normally distributed. The flow diagram of study selection is shown as Fig. 1.

### Risk of bias in the included studies

**Fig. 2:**
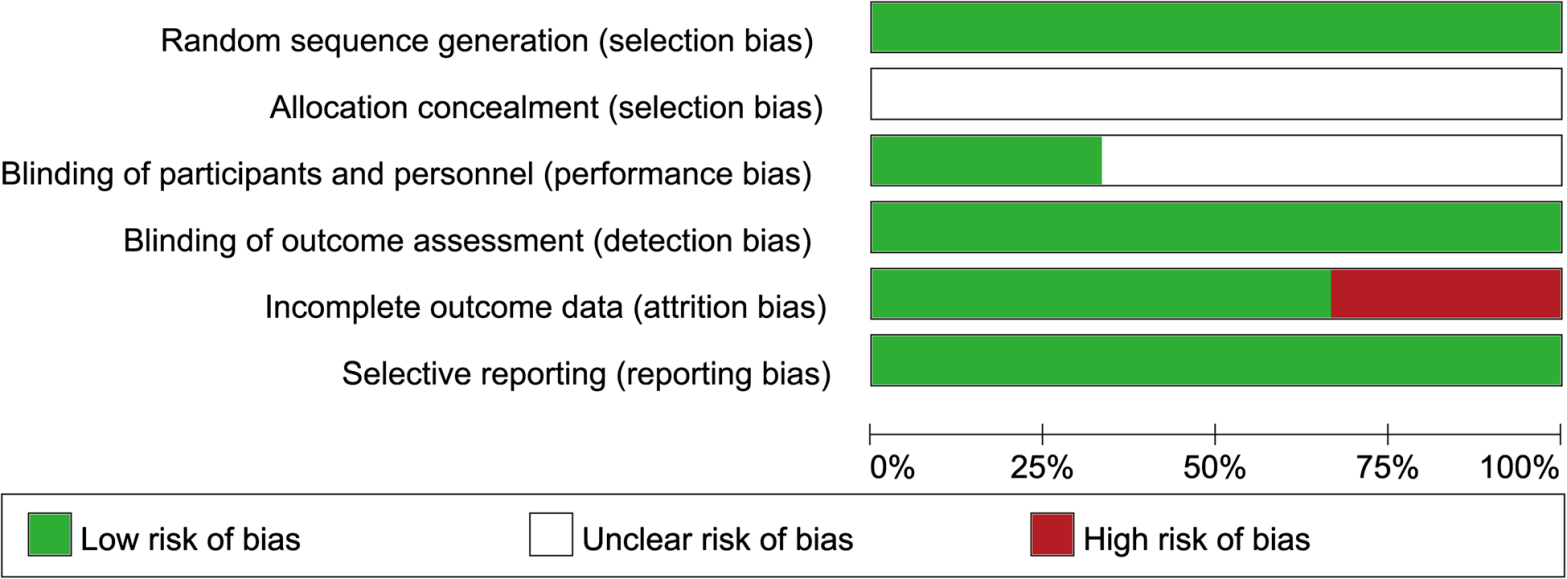
Risk of bias graph. The bias assessment items are presented as low risk, high risk, or unclear risk using different colors

Fig. 2 shows the risk of bias assessments for the included studies. All the trials had reported the random sequence generation, but were determined with an unclear risk of allocation concealment. Only one study reported the blinding procedure of the participants and personnel in detail [33]: “All of the patients were naive to both TMS and partial SD; and thus, it was not likely that they could recognize the treatment modality”. The other two studies had unclear or high risk of bias with respect to the procedures of the participants and personnel. One study had a high risk of incomplete outcome data [32]. The blinding of outcome assessment and selective reporting in the included trials were all of low risk. No other biases had been reported in the three included studies.

### Basic information of the included studies

#### Characteristics of the included studies

The characteristics of the included studies are shown in Table 1. Except for four dropouts in the active/sham rTMS treatment procedures without giving any reasons [32], 72 depressed patients with complete treatment and measurement were finally included in this systematic review. The primary diagnoses of the patients in the three studies were major depression and bipolar disorder. The patients received TSD [32] or PSD [30, 33] in combination with rTMS treatment in all of the studies. For TSD, the patients were deprived of sleep for a period of 24 h from 8:00 pm until 8:00 pm the next evening. In contrast, for PSD, the patients went to bed as usual and were woken up at 1:30 am. Then, they remained awake until 8:00 pm the next evening. All of the subjects received stable pharmacotherapy throughout the treatment process. There was no report of adverse events.

**Table 1:** Characteristics of the included studies

Study	Number of subjects (Exp/Ctr)	Mean age, Y (Exp/Ctr)	Gender (F/M)	Treatment strategy	Primary diagnosis	Diagnostic criteria	Presence of pharmacotherapy	Adverse events	Dropouts
Eichhammer 2002	20 (10/10)	46.7 (44.9/48.5)	15/5	PSD + rTMS	15 Major Depression; 5 Bipolar Ι	DSM-IV	Stable Pharmacotherapy	—	0
Kreuzer 2012	37 (21/16)	43.0 (45.3/39.9)	19/18	TSD + rTMS	Acute Depression	DSM-IV	Stable Pharmacotherapy	—	4
Krstić 2014	19 (11/8)	48.3 (—/—)	19/0	PSD + rTMS	Unipolar Major Depression	DSM-IV and MINI	Stable Pharmacotherapy	—	0

*Note*: Ctr represents control group; *DSM-IV* Diagnostic and Statistical Manual of Mental Disorders, 4th Edition, *Exp* experimental group, *F* female, *M* male, *MINI* Mini-International Neuropsychiatric Interview Criteria, *PSD* partial sleep deprivation, *rTMS* repetitive transcranial magnetic stimulation, *TSD* total sleep deprivation, *Y* years; and a dash (—) indicates no report in the article

#### rTMS parameters

The included studies used different rTMS interventions (Table 2). One of the trials used ten sessions of LF-rTMS (1.0 Hz) over the right DLPFC for a total of 3,000 pulses [33], and the other two used HF-rTMS (10.0 Hz) over the left DLPFC, with four sessions of 4,000 pulses [30, 32]. The intensity varied between the different trials. Moreover, the sham rTMS control was conducted by using a sham figure eight-shaped coil [30, 32] or by applying a real coil perpendicular to the scalp [33].

**Table 2:** rTMS parameters used in the included studies

Study	Frequency (Hz)	Intensity (% of MT)	Sessions	Total pulses	Coil/position	Arm of sham rTMS (Yes or No)	Method of sham rTMS
Eichhammer 2002	10.0	80	4	4,000	Figure eight-shaped / Left DLPFC	Yes	Sham Coil
Kreuzer 2012	10.0	110	4	4,000	Figure eight-shaped / Left DLPFC	Yes	Sham Coil
Krstić 2014	1.0	110	10	3,000	Figure eight-shaped / Right DLPFC	Yes	Coil, Perpendicular to the Scalp

*Note*: DLPFC represents the dorsolateral prefrontal cortex; *Hz* periods per second, *MT* motor threshold, *NR* no report of the method of sham rTMS in the article, and *rTMS* repetitive transcranial magnetic stimulation

### Primary outcome measures

In Eichhammer’s article [30], the data were expressed as means and a box plot diagram. In the diagram, the medians and interquartile ranges as well as the maximums and minimums were shown. From the means and the box plot diagram, it was evident that the outcome data of this trial were skewed. In their paper, Eichhammer et al. [30] reported a stabilizing antidepressant effect of rTMS for SD. The patients were designed to receive one night of PSD during four days of rTMS treatment. On the day prior to the PSD (day 0), the HRSD scores in both groups were comparable (active rTMS, 27.7 ± 5.5; sham rTMS, 26.9 ± 7.3). After PSD (day 1), little difference was observed between the active and sham groups (HRSD scores of 9.1 ± 5.6 and 9.4 ± 4.2, respectively). However, after completing four sessions of rTMS treatment (day 4), the mean HRSD score of the PSD responders in the active rTMS group remained at 9.0 (median, 9.0; interquartile range, 2.0 ~ 16.0), while it went up to 19.0 (median, 20.0; interquartile range 13.0 ~ 24.0) in the sham group.

The other two studies reported normally distributed data. Among them, Kreuzer’s article [32] did not directly express the outcome measures as the mean value ± the s.d. but provided the initial data of each subject instead. Therefore, the post-treatment mean values and s.d. of the groups were calculated based on the original data. The calculations excluded the drop-out patients, and the outcome was consistent with the line chart provided by the authors in the article. The primary outcome measures of these two studies are summarized in Table 3, including the number of subjects involved in the calculation and the mean and s.d. of the HRSD values. A bit difference was found between the two studies. In Kreuzer’s article, it indeed showed a decrease of HRSD score in the experimental group (real rTMS added to SD) when compared with the control (sham rTMS added to SD), but the decrease was not significant. However, in Krstić’s study, the HRSD scores in the experimental group were significantly lower than that in the control group.

**Table 3:** Summary of the primary outcome measures

Study	Outcome measures	N_Exp_/N_Ctr_	MV_Exp_/MV_Ctr_	s.d._Exp_/s.d._Ctr_
Kreuzer 2012	HRSD Scores	19/14	8.74/10.5	6.09/5.52
Krstić 2014	HRSD Scores	11/8	17.5/23.9	5.6/3.8

*Note*: Ctr represents control group, *Exp* experimental group, *HRSD* Hamilton rating scale for depression, *MV* mean value, *N* number of subjects involved in the systematic review, and *s.d*. standard deviation

### Secondary outcome measures

Apart from the HRSD scores, two studies included in this systematic review also reported a secondary rating scale for the antidepressant effects of SD and rTMS. Krstić’s study reported CGI scores [33]. And in Kreuzer’s article, BfS scores, which were suitable for the evaluation of rapid mood changes, served as a subjective efficacy measurement [32]. The secondary outcome measures are summarized in Table 4. Both the CGI and the BfS scores showed good improvement of depressive symptoms in the experimental group.

**Table 4:** Summary of the secondary outcome measures

Study	Outcome measures	N_Exp_/N_Ctr_	MV_Exp_/MV_Ctr_	s.d._Exp_/s.d._Ctr_
Kreuzer 2012	BfS Scores	19/14	23.61/18.71	13.57/10.38
Krstić 2014	CGI Scores	11/8	3.00/3.78	0.89/0.67

*Note*: BfS represents self-reported well-being scale; *CGI* clinical global impression scale, *Ctr* control group, *Exp* experimental group, *HRSD* Hamilton rating scale for depression, *MV* mean values, *N* number of subjects involved in the systematic review, and *s.d.* standard deviation

### Follow-up effects

A total of 50 subjects were enrolled in the follow-up assessment. The follow-up results of the primary and secondary outcome measures are shown in Table 5 and Table 6. The data show a maintained result with the immediate effects of the combined use of rTMS and SD.

**Table 5:** Follow-up of the primary outcome measures

Study	Outcome measures	Follow-up duration (wk)	N_Exp_/N_Ctr_	MV_Exp_/MV_Ctr_	s.d._Exp_/s.d._Ctr_
Kreuzer 2012	HRSD Scores	1	18/14	12.29/13.57	8.12/7.65
Krstić 2014	HRSD Scores	3	10/8	16.7/25.2	5.7/4.5

*Note*: Ctr represents control group; *Exp* experimental group, *HRSD* Hamilton rating scale for depression, *MV* mean values, *N* number of subjects involved in the systematic review, *s.d.* standard deviation; wk, week

**Table 6:** Follow-up of the secondary outcome measures

Study	Outcome measures	Follow-up duration (wk)	N_Exp_/N_Ctr_	MV_Exp_/MV_Ctr_	s.d._Exp_/s.d._Ctr_
Kreuzer 2012	BfS Scores	1	18/14	25.07/22.33	16.28/15.13
Krstić 2014	CGI Scores	3	10/8	2.55/3.89	0.93/0.78

*Note: Ctr represents control group; Exp* experimental group, *HRSD* Hamilton rating scale for depression*, MV* mean values, *N* number of subjects involved in the systematic review, *s.d.* standard deviation, *wk* week

## Discussion

Although numerous systematic reviews and/or meta-analyses have been published and proved that rTMS was an effective treatment for depressive patients [52–56], there were few reviews addressing the combined therapy of rTMS with other antidepressant treatments. Only one recent meta-analysis conducted by Liu et al. [57] had reported an augmentative effect of rTMS on medication treatment for medication-resistant depression patients. Nevertheless, the study of Liu et al. did not concentrate on the combined use of rTMS and SD. In this work, we reviewed the literature on the effects of SD and rTMS co-therapy for depression and attempted to determine whether rTMS could augment the antidepressive effect of SD. The publications showed different changes of depressive symptoms in the depression patients of the experimental group (SD + active rTMS) compared with the subjects of the sham control (SD + sham rTMS). The different primary outcome measures in Kreuzer’s and Krstić’s articles might result from the different SD strategies and rTMS parameters used in the treatment process. Currently, late PSD (in the second half of the night) has been considered to be as effective as TSD. However, doubts have also been expressed by others who consider TSD to be superior [58–60]. In Kreuzer’s study [32], TSD was used prior to the sham or active rTMS. The results showed an obvious improvement in depressive symptoms following TSD, and this improvement lasted throughout the treatment period of rTMS, regardless of sham or active rTMS, which resulted in a longer-lasting antidepressant effect of TSD in the sham rTMS group. Besides, LF-rTMS and HF-rTMS might have contributed differently to depression patients. However, up to now, no other evidence has been available to differentiate the antidepressant effects between PSD and TSD, or between LF-rTMS and HF-rTMS. Therefore, further studies are needed to verify these inferences.

Depression can be associated with various factors, such as hyperglycemia [61], obesity [62], stroke [63], cardiovascular disorders, stress, and anxiety. According to Martinac’s review [64], hyperactivity of the hypothalamic-pituitary-adrenal axis and changes in the immune system are characteristics of depressive disorders. Huang et al. [65] also suggested that depression-like behaviors in rodent models and in humans could be improved by enhancing the brain N-methyl-D-aspartate function. To date, the mechanisms of the augmented effect of rTMS on SD have not been clarified. Krstić et al. concluded in their article that the combined use of rTMS and PSD or the use of either treatment alone would affect the same cortical regions with a possibly synergistic effect [34]. Moreover, the metabolic activity of the anterior cingulate cortex was considered important for the antidepressant action of both SD and rTMS, which could contribute to the enhanced efficacy of SD + rTMS co-therapy [30, 32]. Further animal model studies of depression with rTMS and SD co-therapy may help to understand the underlying mechanism.

Certain limitations exist in this systematic review. First, the outcome measures in Kreuzer’s study were calculated according to the original materials provided [32], which might lead to errors with the measurements. Second, potential publication bias might be an influence factor for the power of this systematic review. Third, the included RCTs did not report the methods of allocation concealment, which induced an unclear risk of bias for the study. Finally, the present review contained only three articles from two research groups so that the total sample size was too small to accurately predict the effect of rTMS on SD. More RCTs with larger sample sizes are warranted.

## Conclusions

From this systematic review, an overview of the research for the combined use of rTMS and SD is presented. Only three studies are now available in the databases. Two of them showed augmentative effects of rTMS on SD in depressive patients; while the other one showed non-significant effects. The few publications limited a conclusive evidence for the therapeutic effects of SD and rTMS co-therapy in patients with depression. However, these studies provide a direction for future research of rTMS-SD co-therapy for depression. Further well-designed studies with a larger number of patients are highly warranted to confirm whether there is an augmentative antidepressant effect of rTMS on SD.

## Additional file

Additional file 1:
**S1 Checklist.** PRISMA 2009 Checklist. (PDF 201 kb)

## Abbreviations

BfS: self-reported well-being scale
CGI: clinical global impression rating scale
CI: confidence intervals
Ctr: control group
DLPFC: dorsolateral prefrontal cortex
Exp: experimental group
F: female
HF-rTMS: high-frequency repetitive transcranial magnetic stimulation
HRSD: hamilton rating scale for depression
Hz: periods per second
LF-rTMS: low-frequency repetitive transcranial magnetic stimulation
M: male
MADRS: Montgomery-Åsberg Depression Scale
MT: motor threshold
MV: mean values
N: number of subjects
NR: no report of the method of sham repetitive transcranial magnetic stimulation in the article
PSD: partial sleep deprivation
RCTs: randomized controlled trials
rTMS: repetitive transcranial magnetic stimulation
s.d.: standard deviation
SD: sleep deprivation
TMS: transcranial magnetic stimulation
TSD: total sleep deprivation
WMD: weighted mean differences
Y: Years
